# Yellow Fever at New Orleans

**Published:** 1848-04

**Authors:** 


*Yellow Fever at New Orleans.*—The late epidemic was probably the most extensive that ever prevailed in this city. As to its severity and mortality, there may be difference of opinion. It is impossible to make a correct computation of the whole number of cases. Some have estimated it as high as *twenty* or *twenty-five thousand*, but we are inclined to think either of these calculations above the truth. As to the mortality, the reports from the cemeteries, as well as they could be obtained from the Board of Health, only make out something upwards of 2300 from yellow fever; but this again is thought by many to be far short of the reality. It is much to be regretted that we cannot obtain greater precision in such important details. The fever raged as an epidemic about two months, and the greatest mortality from it was in September, when the number of deaths reported to the Board of Health, amounted to 1044. During the prevalence of yellow fever in this city, the most frightful and exaggerated reports circulated abroad, but we shall really never get at the truth, unless greater efforts be made than any hitherto exercised.—*N. Orleans Med. and Surg. Jour.*

